# Immunogenic Cell Death: A Step Ahead of Autophagy in Cancer Therapy

**DOI:** 10.33696/cancerimmunol.3.041

**Published:** 2021

**Authors:** Gourab Gupta, Kristina Borglum, Hexin Chen

**Affiliations:** Department of Biological Science, Center for Colon Cancer Research, University of South Carolina, Columbia, SC 29208, USA

**Keywords:** Autophagy, Apoptosis, Immunogenic cell death, Caspase, ATP, Multi-drug resistance

## Abstract

Immunogenic cell death (ICD) plays a major role in providing long lasting protective antitumor immunity by the chronic exposure of damage associated molecular patterns (DAMPs) in the tumor microenvironment (TME). DAMPs are essential for attracting immunogenic cells to the TME, maturation of DCs, and proper presentation of tumor antigens to the T cells so they can kill more cancer cells. Thus for the proper release of DAMPs, a controlled mechanism of cell death is necessary. Drug induced tumor cell killing occurs by apoptosis, wherein autophagy may act as a shield protecting the tumor cells and sometimes providing multi-drug resistance to chemotherapeutics. However, autophagy is required for the release of ATP as it remains one of the key DAMPs for the induction of ICD. In this review, we discuss the intricate balance between autophagy and apoptosis and the various strategies that we can apply to make these immunologically silent processes immunogenic. There are several steps of autophagy and apoptosis that can be regulated to generate an immune response. The genes involved in the processes can be regulated by drugs or inhibitors to amplify the effects of ICD and therefore serve as potential therapeutic targets.

## Introduction

Cell Death has long been considered to be an inevitable part of the life cycle of a cell and hence, considered a familiar consequence of cellular life. However, accumulating evidences regarding regulated cell death (RCD) changed this concept and drew enormous attention to this field. RCD is the process by which redundant, irreversibly damaged, or malfunctioning cells are removed from the system in an organised homeostatic driven manner. RCD can be triggered by either intrinsic and extrinsic processes, depending on the amount of stress or damage induced in the cells. RCD occurring from intrinsic physiological conditions is termed as programmed cell death (PCD). The effects of RCD are not constricted to an individual cell. Rather, it encompasses the entire system that helps to maintain homeostasis [1]. RCD requires the involvement of a tightly regulated signalling cascade. Regulated cell death induced by external factors can also be termed as RCD. Chemotherapeutic drug induced tumor cell death is mediated by PCD, which includes the process of apoptosis or autophagy. However, these mechanisms of cell death are generally not immunogenic. In order to invoke an immunogenic form of cell death, which involves tumor associated antigen presentation to immune effector cells and immune cell mediated killing of cancer cells, we need to induce immunogenic cell death (ICD). In this review we will look at various strategies that can be employed to get an immunogenic form of apoptosis and autophagy as well as improve the effects of drug induced ICD.

## Types of Programmed Cell Death

There are three different types of common PCD: apoptosis, necrosis, and autophagy. Apoptosis can be triggered by both intrinsic and extrinsic pathways that involve the formation of apoptotic bodies that contain fragmented cellular compartments, including DNA, and are eliminated by phagocytosis. In general, apoptosis does not tigger inflammation. Necrosis is characterised by disruption in the cell membrane, swelling of cytoplasm and mitochondria, and the breakdown of organelles. DNA is degraded randomly by extracellular DNAse I or by lysosomal DNase II. During necrosis, the cellular contents are released in the extracellular space acting as danger signals, therefore inducing inflammation [2]. Autophagy or macroautophagy is a conserved catabolic process by which cells try to get rid of unwanted substances by packaging them into autophagosomes and degrading those using lysosomal enzymes, thus maintaining homeostasis. Autophagosomes containing cargo fuse with lysosomes forming autolysosomes, which leads to degradation of the materials present inside and the release of amino acids and fatty acids which are used to meet the bioenergetics requirements of the cell. PCD is an immunologically silent phenomenon as it is required for the normal functioning of the cell and the maintenance of tissue homeostasis. In order to attract immune cells to the tumor microenvironment (TME), we need to trigger ICD.

## Immunogenic Cell Death: A Peculiar Form of RCD

An important aspect of cancer cell survival involves the various strategies that they employ to evade immune response and create an environment that supports their proliferation. In order to cope with these measures, our body has various intrinsic mechanisms to deal with them. If not sufficient, some external drugs have also been developed that help induce immunogenic response against the tumor from signals generated by the tumor cells themselves. ICD is one such form of RCD which depends on the chronic exposure of tumor associated Damage Associated Molecular Patterns (DAMPs) to the immune system helping drive autoimmunity and generate antitumor response in the TME [3]. ICD helps in turning dying tumor cells to vaccines, which induce antitumor immunity by DC maturation cytotoxic T cell activation [4].

Apoptotic cell death is a very common phenomenon and is regularly happening in our body to get rid of defective cells, as well as maintain tissue homeostasis. Because of the vital role of apoptosis for the regular functioning of the body, it is not immunogenic. Therefore, cancer cells undergoing apoptosis do not induce an immune response. In addition, immunosuppressive compounds, like Transforming Growth Factor-β (TGF-β), released during the course of apoptosis also down regulate the immune response. Autophagy is also related to cell death or cell defence against stress response. However, its immunological characteristic is still not well defined. Autophagy is most commonly used by cancer cells for evading an immune response. Therefore, it is clear that these cell death procedures are not sufficient to invoke immune cells to actively fight off a tumor. In order to activate the immune system, it is first necessary to point out the cancer cell mediated release of compounds to be pathogenic and recruit APCs to actively pick up tumor antigens for presentation to the cytotoxic T cells.

The commencement of ICD depends largely on the stress developed in the Endoplasmic Reticulum (ER) and the increased level of Reactive Oxygen Species (ROS) due to various ICD inducers [5]. ER stress is basically generated due to formation of misfolded or unfolded proteins in the ER that start aggregating in the ER, inducing ER stress mediated signalling. In order to cope with excessive ER stress, cells initiate the Unfolded Protein Response (UPR) mechanism which stalls translation, thereby limiting protein synthesis and relieving the cell of the ER stress. However, this occurs only when the ER stress is mild enough to mitigate the effects by reducing protein synthesis. Further increase in the amount of ER stress leads the cell to apoptosis or ICD based on the inducer of ER stress. The ultimate outcome of this overstressed environment is the release of DAMPs on the cell surface or in the TME which are true danger signals that alert the immune system to take required action, as well as alert the cellular community to the induced stress response.

## Major Hallmarks of ICD

Taking a closer look at DAMPs, they are intracellular molecules which are expressed on the cell surface or released by the cell undergoing stress. They have immune-stimulatory effects like DC maturation, Tumor antigen presentation, activation of cytotoxic T cells, and chemotactic effects on innate immune cells. DAMPs can be broadly categorised into 3 types depending on their stage and localisation/release place: 1) DAMPs appear on the cell surface (e.g., CRT, HSP70 (HSPA), HSP90 (HSPC1)); 2) DAMPs appear extracellularly (e.g., HMGB1, uric acid and pro-inflammatory cytokines); and 3) DAMPs appear as end-stage degradation factors (e.g., ATP, DNA and RNA).

**Figure 1:**
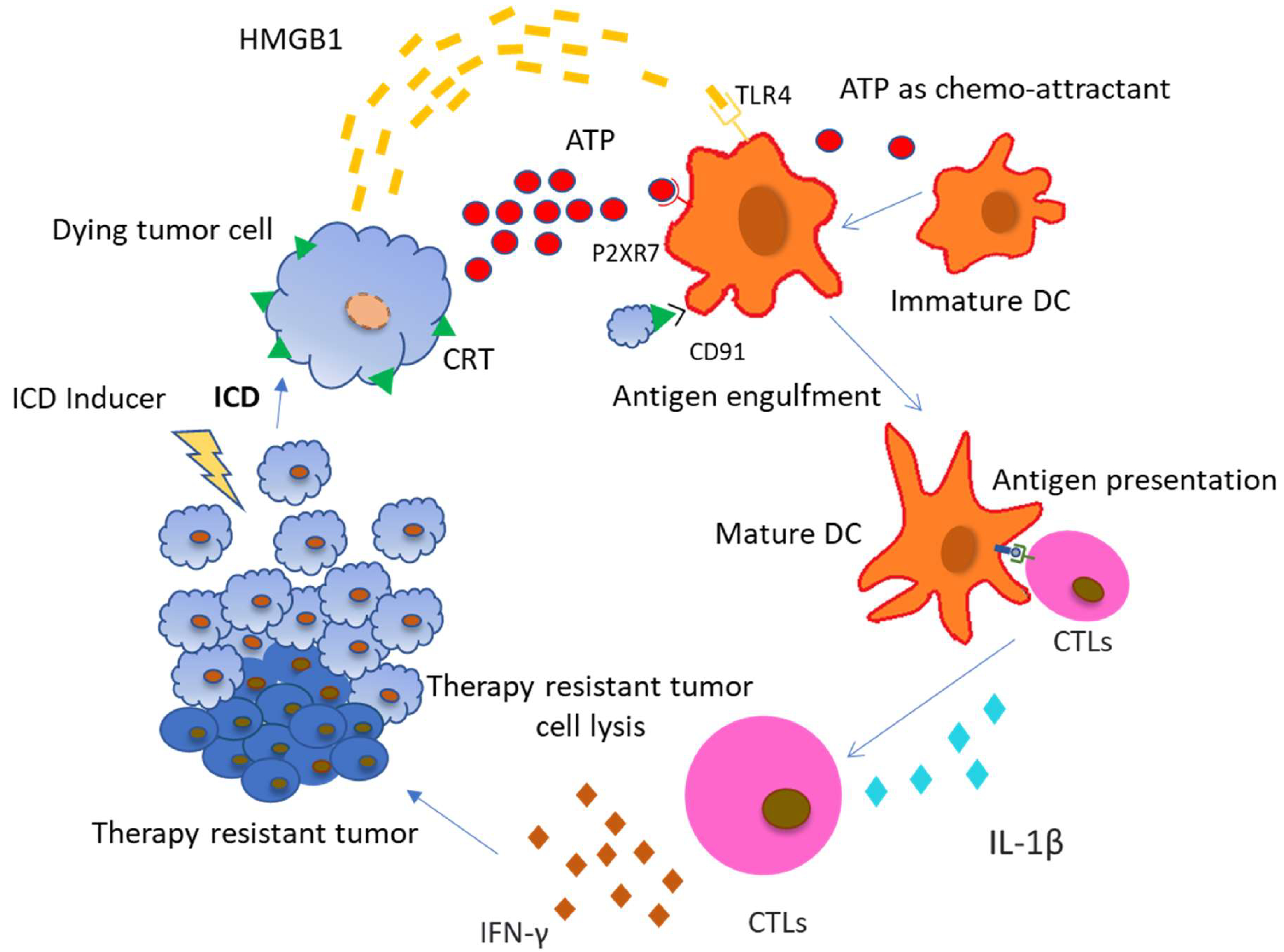
Chemotherapeutic drugs inducing Immunogenic Cell Death (ICD) in tumor cells mediate the release of DAMPs from the dying tumor cells. Cancer cells dying due to ICD induce CRT exposure on the outer surface of plasma membrane and secrete ATP and HMGB1. These DAMPs have respective receptors on various immune cells. Most commonly, Antigen Presenting Cells (APCs) are the ones that are first recruited to the site of ICD and pick up signals to start an immune response. They contain TLR4, P2X7R and CD91 receptors on their surface which recognise HMGB1, ATP and surface exposed CRT respectively. ATP helps in the recruitment of DC to tumor bed, CRT helps in the uptake of tumor antigens by DCs and HMGB1 helps in optimal antigen presentation to T cells. Cytotoxic T lymphocytes (CTLs) are activated by these mature DCs by antigen presentation and IL-1β secretion. CTLs produce inflammatory cytokines like IFN-γ which leads to the elimination of chemotherapy resistant tumors. (Abbreviations: ICD: Immunogenic Cell Death; CRT: Calreticulin; ATP: Adenosine Triphosphate; HMGB1: High mobility group Box 1; APCs: Antigen Presenting Cells; TLR4- Toll-like receptor 4; IL-1β- Interleukin 1 beta; IFN-γ: Interferon-gamma; CTL: Cytotoxic T lymphocyte).

Calreticulin (CRT) has one of the most important roles in promoting ICD induced antitumor response and is directly targeted by ER stress related signalling. CRT binds to CD91 receptors on DCs, enabling phagocytosis of dying cancer cells as well as antigen cross-presentation to CTLs (Figure 1). CRT-CD91 interaction triggers the NF-κB signalling pathway in DCs and helps release a series of proinflammatory cytokines in the extracellular matrix, leading to Th17 priming [6]. Heat-Shock proteins (HSP) residing in the ER lumen or cytoplasm are translocated to the cell surface during ER stress condition or during exposure to chemotherapeutic substances. HSPA, HSPC1, and Gp96 (HSPC4) are involved in ICD. Ecto-HSPA and HSPC4 function as DAMPs, as they can interact with the APCs like CD91, LOX1, and CD40 [7]. HSPC4 is another type of HSP that is involved in tumor antigen presentation to the MHC class I molecule, activating a CD8+ T cell response [8]. ATP and HMGB1 are other important DAMPs which are discussed later. CRT exposure, ATP secretion, and HMGB1 release are all indispensable for ICD and the absence of any one is likely to render chemotherapy based cytotoxicity to tumor cells as ineffective. We are going to discuss in detail the role of ATP in connecting autophagy and ICD.

## Role of ATP in Connecting Autophagy and ICD

Pre-mortem autophagy is required for the ICD associated secretion of ATP, implying that cancer cells deficient in carrying out the process of autophagy fail to produce therapy induced immune response in vivo. However, pharmacological activation of autophagy is not sufficient to induce an immunogenic response [9]. The process of autophagy plays an important role in the release of ATP, which in turn is a necessary step in the induction of ICD. It has been well studied in autophagy-deficient (Atg5^KD^ or Atg7^KD^) CT26 tumor cells. Mass spectrometric analyses were performed to look for immunogenic signals in the supernatants from mitoxantrone (MTX)-treated CT26 colorectal carcinoma cells. It was found that autophagy deficient tumor cells released lower amounts of ATP compared with their autophagy-competent counterparts. Interestingly, autophagy deficient cells as well as autophagy-competent cells showed similar exposure of CRT and release of HMGB1 upon treatment which establishes a direct link between autophagy and ATP release [10].

The ATP released from dying cells acts as a prominent “find me” signal for immature macrophages and DCs upon binding to the P2Y_2_ receptors of myeloid cells [11,12]. Apart from that, the ATP released from dying cells also activates P2RX_7_ receptors on DCs which stimulates the NLRP3 inflammasomes, a caspase-1 activation platform, which further stimulates the cleavage of pro–IL-1β and the release of IL-1β, which is most important for priming of IFN-γ–producing, tumor antigen–specific CD8+ T cells. Furthermore, it was also confirmed experimentally that DCs derived from the bone marrow of *Casp1^−/−^* or *Nlrp3^−/−^*mice, pulsed with dying tumor cells, failed to elicit CTL mediated antitumor effects, suggesting that ATP- P2RX_7_ induced NLRP3 mediated caspase-1 activated IL-1β production is crucial for the IFN-γ–producing T cells to produce antitumor effects [13].

Evidences also suggest that P2RX7 receptors present on leukemic cells are activated with increasing concentration of extracellular ATP and can lead to cancer cell death by mitochondrial membrane damage and caspase-3 activation [14]. Increased release of ATP by drug induced ICD in itself is toxic to the cancer cells and induces further apoptosis in a caspase dependent manner. Apart from direct effects, extracellular ATP is also involved in communicating with other immune cells to regulate tumor growth. Tumor associated macrophages are key regulators of the immune response in the TME. Based on their polarization, macrophages have different roles in mediating immune response against tumors. M1 macrophages are involved in a pro-inflammatory response via the NLRP3 proinflammatory inflammasome pathway when there is an increase in extracellular ATP concentration [15]. Apart from macrophages, Dendritic cells (DCs) are also affected by an increase in extracellular ATP concentration. P2X7R and P2Y11R receptors on DCs bind to extracellular ATP, promoting chemotaxis and differentiation of immature DCs [16]. DC activation by binding to ATP also promotes caspase-1 dependent NLRP3 inflammasome formation and the release of IL-1β [16], which in turn promotes CD8^+^ T cells [16] and IL-17 [17] producing-γδ T cell mediated anti-tumor response. Apart from that, IL-1β is also involved in the production of IFN-γ from γδ T cells and CTLs, which are responsible for the elimination of therapy resistant cancer cells [18]. Purinergic receptors (like P2X7) are also found on T cells, which are activated by an increasing concentration of extracellular ATP in the TME. Accompanied by an increased influx of Ca^2+^, activated T cells release IL-2 which is involved in further activation of CD8^+^ and CD4^+^ T cells [19], as well as memory T cells. Removal of extracellular ATP or silencing of the P2X7 receptor inhibits Ca^2+^ entry and activation of T cells [20].

Therefore, sufficient release of ATP in the extracellular environment is critical for an immunogenic response. There are several therapies targeting the levels of extracellular ATP and the purinergic receptors that are important for boosting anti tumor immunity. Several defects in the cancer cells might prevent the accumulation of ATP in extracellular spaces, which could have an immunosuppressive role on the tumor. One such defect is epigenetic changes in the molecular machinery of autophagy, leading to poor efflux of ATP from dying tumor cells [21] and another defect being the overexpression of ectonucleoside triphosphate diphosphohydrolase 1 (ENTPD1, best known as CD39) or 5'-nucleotidase, ecto (NT5E, best known as CD73) which are membrane-bound nucleotidases that degrade extracellular ATP [22] to ADP, AMP, adenosine, and inorganic phosphate. Extracellular adenosine acting at the A2A receptor may be involved in down-modulating the immune response, as it is found in elevated levels in the TME [23]. These factors prevent the generation of an immune response in spite of an autophagy induced efflux of ATP.

A favourable means by which this can be prevented is by administration of a broad spectrum inhibitor of extracellular nucleotidases, ARL67156. It has been shown that CT26 cells engineered to overexpress CD39 and exposed to anthracyclines are unable to prevent tumorigenesis in syngeneic tumor models [24]. Co-administration of ARL67156 along with anthracyclines helped maintain the ATP pool in the extracellular environment, which is required for the activation of the immune response and progression to ICD [10]. Thus, it can be said that the use of extracellular nucleotidases inhibitors along with ICD inducers help in increasing the efficiency of these drugs and in maintaining the ATP build-up in the extracellular space, which is vital for the signalling of DCs and Cytotoxic T cells to launch an immune response against the tumor. Another strategy to prevent the degradation of extracellular ATP is by using biocompatible and biodegradable nanoparticles loaded with ATP, which can provide a controlled release of ATP in the TME and enhance the tumor cell cytotoxicity of this nucleotide [25].

P2X7 receptors on immune cells play a major role in their activation and their response to tumor cells. Positive allosteric modulators like polymyxin-B, clemastine, and ginsenosides can be used to enhance P2X7 mediated cytotoxicity against cancer cells [26–28]. Another strategy developed by Igawa and co-workers utilizes the increased extracellular ATP levels in TME to induce immunogenic response by means of anti CD137 antibodies. They developed anti CD137 switch antibodies (STA551) that elicit its agonistic activity only in the presence of high ATP concentration (>100 micromol/L), similar to that present in TME in ICD conditions. When synergistically used with anti PD-1, this antibody showed increased CD8+ T cell proliferation and tumor infiltration. This proves that it can be used as a potential co-treatment strategy to boost antitumor immune response [29].

We have previously discussed the activation of cytotoxic T cells by an increased concentration of extracellular ATP levels in TME. However, recent studies have shown that chemotherapeutic drugs inducing an ICD mediated increase of ATP also lead to the activation and maturation of T_regs_, which has an immunosuppressive effect. Daunorubicin (a chemotherapeutic drug) was found to stimulate antitumor T-cell response in solid tumors and AML, but the treatment was not curative, indicating that there was a missing link in the treatment. It was shown from AML patient sample studies that apart from CTL activation, elevated ATP levels also lead to the co-activation of a new set of T_regs_ that have immunosuppressive function. This subset of T_regs_ also showed higher expression of the immune checkpoint inhibitor PD-1 [30]. PD-1 expression on the T_regs_ is the reason for their immunosuppressive activity [31,32]. Anti-PD-1 antibodies like pembrolizumab or nivolumab in combination with Daunorubicin can increase the ICD mediated cytotoxicity of tumor cells by supressing T_reg_ function, as well as promoting effector T cells activation.

## Role of Caspase-3 in ATP Release and Induction of ICD

The previous discussion referred to the importance of autophagy induced ATP secretion for the generation of ICD. However, it is well understood that in spite of being important, simple induction of autophagy is not enough for eliciting an immune response [33]. Moreover, it has also been shown that autophagic cell death is crucial for ATP secretion. Autophagy promoting drugs that fail to trigger cell death are unable to induce sufficient ATP secretion to generate an immune response. Thus, it can be concluded that autophagy alone is not sufficient to elicit an ICD-associated release of ATP. Studies demonstrated that a fine regulation between caspase mediated apoptotic cell death and autophagy must take into account for sufficient ATP secretion in the course of ICD [34]. A close connection between the apoptosis machinery and the autophagic machinery that is being discovered might lead to a situation where simultaneous activation of the processes would lead to the observable outcome.

Pannexin 1 (PANX1) channels play a crucial role in the release of ATP from apoptotic cells and require caspase-3 to cleave at the C-terminal auto-inhibitory domain to produce the active form, tPANX1. The role of PANX1 and caspase-3 in the release of ATP from apoptotic cells has been confirmed by the pharmacological inhibition of these proteins by knockouts and deletions in cell lines [35–37]. It has been shown that MTX treated ATP secretion was inhibited when tumor cells were treated with broad spectrum caspase inhibitors. Upon cleavage by caspase-3, PANX1 forms channels across the plasma membrane which allows for the free diffusion of molecules like ATP. Caspase activation pathway is triggered by the ligation of a transmembrane death receptor in response to extracellular stimuli or the release of mitochondrial death factors. These events first activate initiation caspases, such as caspase-8, −9 and −10, which further cause the downstream activation of caspase-3, −6 or −7 [38]. Activation of the extrinsic and/or intrinsic apoptotic pathway sensitises the tumor cells to the cytotoxic stimuli. Administration of most anticancer drugs like doxorubicin in breast cancer [39], methotrexate in lymphoid leukemias [40], or bleomycin in hepatocellular carcinoma [41], activates the apoptotic caspase cascade. However, many of chemotherapeutic drugs are not efficient to trigger ATP release.

It turns out that ATP does not distribute evenly within the cell. Interestingly, in some drug-treated cells, ATP colocalizes with LAMP1, a lysosome membrane protein and can be co-stained with lysosomotropic dyes as well [18]. Upon induction of autophagy, ATP is also found to co-localize with the MAP1LC3 protein, which is critical for the biogenesis of autophagosomes. At a later stage in apoptosis, LAMP1 and phosphatidylserine are expressed on the cell surface, confirming the fusion of autolysosomes with the plasma membrane and the subsequent release of the cargo, which includes ATP, in the extracellular microenvironment. This establishes the link between caspase, PANX1, and autophagy in the release of ATP for induction of ICD [18].

**Figure 2:**
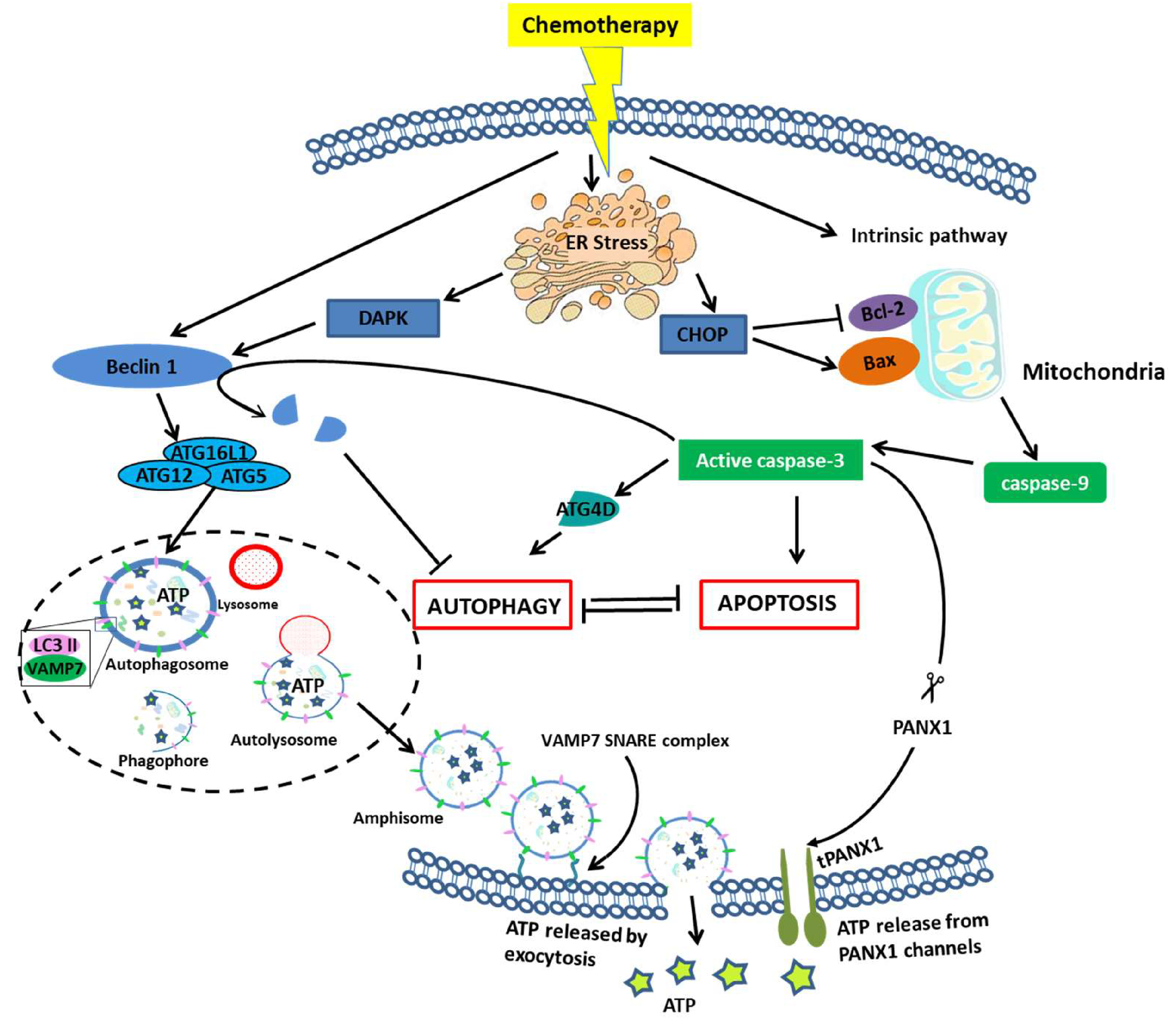
Crosstalk between autophagy and apoptosis leading to the release of ATP. Continued ER stress induced by chemotherapeutic agents lead to cell death by either autophagy or apoptosis. There is a mutual inhibition mechanism between apoptosis and autophagy. The intrinsic apoptotic pathway can be activated by the release of CHOP due to continued ER stress which activates Bax to induce mitochondrial outer membrane permeabilization (MOMP) and downstream activation of caspase-9. caspase-9 in turn activates effector caspase-3 which brings about apoptosis [43]. However, it has also been found that caspase mediated cleaving of ATG4D helps promote autophagy [44]. The release of ATP in the extracellular space is dependent upon PANX1, which when cleaved by caspase-3 forms channels that help in the release of ATP [37]. Though the release of ATP is dependent on caspase-3 activation, the accumulation of ATP and its transport to the plasma membrane is mediated by the autophagic machinery [18]. ER stress induced phosphorylation of Beclin1 by DAPK and association of Beclin1 complex together with ATG5, ATG12, ATG16L and LC3 II help in the formation of autophagosome [45]. ATP is found to co-localise with these autophagic proteins inside the autophagosome. Fusion of autophagosome with lysosome leads to the formation of autolysosome [46]. Autolysosome matures to amphisome which is a late endosomal complex. ATP loaded amphisome fuses with plasma membrane using the VAMP7-SNARE complex to release ATP in the extracellular medium upon autophagic stimulation [46]. (Abbreviation- CHOP: C/EBP homologous protein; MOMP- Mitochondrial outer membrane permebialization; DAPK: Death-associated Protein Kinase; VAMP7- Vesicle-associated membrane protein 7; SNARE: SNAP receptor; PANX1: Pannexin1).

As we have seen earlier, caspase-3 plays a major role in PANX1 cleavage and activation of the pathway that releases ATP in autophagy induced cells. Hergenrother and colleagues have found a compound that catalyses activation of procaspase-3 to caspase-3. The compound is called PAC-1 and it is the first procaspase-3 activating compound. It has been widely found to induce apoptosis in cancer cells and even promoted tumor growth retardation in mice [42]. This can be used as a co-therapy along with ICD inducing drugs to promote ATP secretion in the extracellular spaces that will help create an increased immunogenic response to tumor cells. However, further experimentation is required to judge its efficacy in the human TME. In order to understand the relation between autophagy and apoptosis and the role of caspase-3 in this, we need to take a look at the earlier steps where the decision of cell death or survival is made. Autophagic cell death machinery is activated in response to an inhibition of apoptotic cell death. ATG proteins are important for both cell death and survival. Investigation in this matter has shown the importance of ATG5 functioning as a switch between autophagic and apoptotic cell death. Cleavage of ATG5 by Calpain induces an apoptotic form of cell death using the autophagic machinery. Therefore, activated ATG5 is necessary for carrying out autophagic cell death. Further investigation has also revealed that ATG6 acts as a suitable target of caspase-3 and −6 in order to carry out apoptosis. Downregulation of ATG6 sensitises the cells to apoptotic cell death. The function of ATG6 is regulated by Bcl-2 and PI3K. Bcl-2 not only affects apoptosis by inactivating caspases, but also affects autophagy by inhibition of the ATG6-PI3K complex which is essential for the formation of the autophagosomal membrane (Figure 2). Therefore, a detailed study on the regulation of Bcl-2, PI3K, and ATG6 is important to understand the caspase mediated switching from apoptosis to autophagy.

## Clinical Effects of ICD Induced DAMPs in Cancer Treatment

The clinical relevance of immunogenetic cell death and cancer treatment is very promising, as the methods discussed here used to detect ER stress, calreticulin, HSP, ATP secretion, and caspase-3 activation can be used in identifying and developing new anticancer agents. Many clinical trials have begun in the last ten years to begin determining the efficacy of some approved ICD inducing chemotherapeutics, for example: doxorubicin, epirubicin, idarubicin, MTX, bortezomib, cyclophosphamide, and oxaliplatin [47]. Some of these trials have combined ICD inducers with immune checkpoint blockers (ICBs) or with monoclonal antibodies [1]. Voorwerk et al. conducted a clinical trial in triple negative breast cancer (TNBC) patients where the best objective response rate (ORR) was in patients treated with the ICB, nivolumab, combined with the ICD inducer, doxorubicin [48]. Hiddemann et al. also significantly improved outcomes in patients with follicular lymphoma by combined treatment of doxorubicin with the monoclonal antibody, rituximab [49]. Federico et al. also combined ICDs with a monoclonal antibody, with a very impressive response rate of 61.5% in children with recurrent neuroblastoma and with very strong indications of increased immune activation [50]. A third combination of ICD inducers has been with vaccinations. Kanekiyo et al. combined a peptide vaccine with oxaliplatin treatment in patients with advanced colorectal cancer, resulting in increased cytotoxic T-cell levels and improved outcomes [51]. However, Camisaschi et al. demonstrated a decrease in regulatory T-cells for melanoma patients vaccinated with a peptide vaccine and treated with cyclophosphamide compared with both vaccination alone and the control [52].

While these clinical trials have made it clear that ICD-inducing compounds will play a very important role in the future of cancer therapeutics, the most effective compounds and combinations are still unclear, as shown by the mixed results of superior and inferior prognoses when using ICD inducers in clinical trials. It is also important to note that most clinical trials focus their study on a singular ICD inducer in combination with another therapeutic agent. It will be important to compare the effects of multiple approved ICD inducing compounds when establishing the most effective treatments. This points to the need for further preclinical studies to determine the efficacy of various combinations, as well as identify new ICD inducers.

CRT exposure and HSPA/HSPC4 are important DAMPs that are associated with the activation of APCs to pick up dead cells for processing and presentation of tumor associated antigens. They are also associated with the release of proinflammatory cytokines like IL-6 and TNFα. Clinical studies suggest that patients treated with ICD inducers having higher levels of HSP and CRT exposure have greater chances of survival [53]. Moreover, it has also been found that higher exposure of CRT and HSP tend to attract more CD45RO^+^ memory T cells and can increase the rates of survival by 5 years [54]. HSP enriched cancer cell lysate can be used as an anticancer vaccine to boost antitumor immunity in patients [55]. Another form of antitumor immunity is mediated by type1 interferon production due to TLR3 signalling in anthracycline based drug treated patients. Clinically, TLR3 agonists are only used as prognostic tools to determine risk in cancer patients, as well as predict a future outbreak of cancer in healthy subjects. The role of ATP as a DAMP in ICD mediated cancer therapy has already been discussed earlier in details. HMGB1 release in the extracellular space is a marker for cell death, as it is released only when there is a breakdown of nuclear and plasma membrane [56]. Higher HMGB1 levels in cancer patients with esophageal squamous cell carcinoma has been correlated with increased chances of survival in patients that have undergone chemoradiotherapy [57].

## Strategies to Induce ICD for Overcoming Multi-drug Resistance

Multi-drug resistance rises to be an important factor contributing to the failure of chemotherapy. Resistance of tumor cells to a wide variety of chemotherapeutic drugs can be summarised as multi-drug resistance in cancer cells. Modern advances in genomics, proteomics, and functional analytical techniques have enabled us to gain deeper insights into the genes and signalling pathways involved in multi-drug resistance. It has been shown that acquired multi-drug resistance in cancer cells arise due to the increased expression of therapeutic targets or by activation of alternative compensatory signalling pathways [58] and sometimes due to a high degree of molecular heterogeneity by therapy induced selection of minority resistant population [59]. There are several ways in which this drug resistance is achieved: 1) increased efflux of drugs by ATP dependent transporters [60,61] 2) reducing the absorption of drugs [62] 3) enhancing drug metabolism and elimination by glutathione S-transferase and cytochrome P450 enzymes [63,64] 4) apoptotic pathway blocking by upregulation of anti-apoptotic genes like Bcl2 and AKT [65], along with mutations in the p53 pathway [66] 5) epigenetic regulation and miRNA regulation [67,68] 6) mutation in drug targets [69] 7) changes in microenvironment due to hypoxia [70] or cancer stem cell regulation [71]. One of the above mechanisms involving blockage of apoptotic pathway serves as an important reason for the development of multi drug resistance, as most of the chemotherapeutics work by induction of apoptosis in cancer cells. The focus was then shifted to other forms of programmed cell death, and autophagy was found to be a contributing candidate. Autophagy is basically used as a protective mechanism by tumor cells, which further leads to the development of multi-drug resistance. The role of autophagy in tumorigenesis is already known [72]. However, uninterrupted autophagy may also lead to autophagic cell death, thus autophagy can be suitably used to promote the efficacy of treatment on MDR cancer cells.

The obvious therapy that was initially thought of as a remedy to MDR was inhibition of autophagy as a means to increase sensitisation of cancer cells towards chemotherapy. This includes genetic inhibition of *Atgs,* such as *Beclin1*, *Atg5*, *Atg7*, and *Atg12,* to sensitise MDR cancer cells to therapeutic agents [73,74]. Overexpression of miR-23b-3p or miR-489, which targets autophagy initiating genes, sensitises chemoresistant human cancer cell line to chemotherapeutic drugs [75,76]. However, as already mentioned earlier, uninterrupted autophagy leads to cancer cell death; this can hence be used as a remedy wherein induction of prolonged autophagy induces autophagic cell death in apoptosis deficient MDR cancer cells. There are several anticancer agents that can induce excessive autophagy, thereby killing cancer cells. One of them is suberoylanilide hydroxamic acid (SAHA), a histone deacetylase inhibitor that induces autophagic cell death in tamoxifen resistant MCF-7 breast cancer cells, significantly reducing tumor growth [77]. Therefore, it becomes evident that both inhibition of autophagy or uninterrupted autophagy can be used as a remedy against apoptosis independent MDR cancer cells [78].

However, the question remains whether sensitising MDR cancer cells by regulating autophagy can be made immunogenic for increasing the efficacy of the therapeutic agents. Studies suggest that regulating the premortem autophagic flux can be beneficial in inducing an immunogenic effect by the increased release of DAMPs while undergoing an immunogenic form of cancer cell death [10]. AXL receptor tyrosine kinase mediates multi-drug resistance in cancer cells and supresses tumor immunity. In lung adenocarcinoma, cells develop resistance to EGFRi by acquiring a mutation in the EGFR gene. It was found that the EGFRi resistance was mediated by upregulation of AXL, which apart from inducing MDR was also involved in immune evasion from natural killer (NK) cells and cytotoxic T cells (CTL). Small molecule inhibitors of AXL render MDR cancer cells sensitive to NK cells and CTL [79]. Though this form of treatment has shown fruitful results in tumor growth inhibition and sensitisation of resistant cells, in order to provide long term protection against recurrence and metastasis, an immunogenic form of cell death is necessary. As mentioned earlier, an increased autophagic flux is correlated with the increased expression of AXL and hence can be targeted to modulate the effects of AXL in MDR. It was shown that targeted inhibition of AXL using small molecule inhibitors, like bemcentinib, abrogates the high premortem autophagic flux in resistant cells, thus enhancing immunogenicity by the controlled release of ATP, an increased post-mortem release of HMGB1, and increased expression of CRT on the surface while undergoing an immunogenic form of cell death [80]. AXL signalling abrogates apoptotic pathways and increases autophagic flux, thus inducing multi drug resistant cells. However, increased autophagy does not correlate to increased immunogenicity in this case, but contributes to multi-drug resistance. AXL inhibitors not only sensitise MDR cells to chemotherapy, but also reduce the autophagic flux driving the cells to the release of DAMPs and an immunogenic form of cell death [80]. Another study on colorectal cancer showed a similar outcome when a therapy based on autophagy inhibition and antineoplastic drugs helped in the elimination of drug resistant colorectal cancer cells, as well as developed immunity by DC maturation and CTL recruitment to the TME. It was found that increased autophagy in colorectal cancer cells prevented apoptosis and helped in the development of drug resistance. Using a combination of chloroquine as an autophagy blocker and 5-FU in low concentration helped overcome the drug resistance and induce immunogenic effects in colorectal cancer [81].

The above discussions favor the inhibition of autophagy as a therapy to overcome multi-drug resistance in cancer cells, as well as induce immunogenic cell death. However, the reinitiation of apoptosis in the tumor cells can also be used as a therapy in resistant tumors. Several successful efforts have been taken in this field, including the use of BH-3 mimetics [82]. Their function is to inhibit the Bcl-2 protein and induce apoptosis. A combination treatment of carfilzomib and ABT-263, a BH3 mimetic, significantly improved apoptosis in colon cancer cells with mutant KRAS-mediated apoptosis resistance [83]. Other such BH-3 mimetics include obatoclax, which sensitizes the hypoxic cells to apoptosis induced by 5-FU [84]. Apoptosis induced cell death remains mostly non-immunogenic, but it has been found that ICD inducers often use the apoptotic pathway to increase expression of DAMPs and mediate an immunogenic process of cell death, shikonins being one such example. It activates the apoptotic pathway and increases the release of DAMPs while undergoing ICD [85].

## Conclusion

There lies an intricate balance in the cellular mechanism which determines the outcome of programmed cell death. Tumor cells hijack this pathway for long term survival and immunomodulation. Induction of apoptosis in cancer cells via the use of chemotherapeutic drugs was considered a form of treatment, but the problem with apoptosis lies in the fact that apoptosis does not induce an immunogenic response. Several clinical evidences suggest that the simple use of apoptotic drugs does not provide long term benefits to cancer patients and that there must be some way to activate an immunogenic form of apoptosis [86,87].

We have reviewed the combined pathways of apoptosis, autophagy, and exocytosis required for the efficient release of DAMPs and discussed several ways in which we can improve the immunogenic response to these DAMPs in order to amplify the effects of ICD and overcome chemotherapy resistance. Precisely targeting specific steps of apoptosis and/or autophagy may be more efficient to induce ICD than simply induction or blockage of the entire process. For example, blocking autophagy at a later stage does not affect the distribution of ATP, however it can help prevent the immunosuppressive effect of autophagy, as well as induce ER stress and apoptosis. Similarly, it was found that apoptotic membrane blebbing was required for the efficient release of ATP, but apoptosis is generally not immunogenic. We need to investigate more into the events that are actually involved in the release of DAMPs and try to restrict the events that have immunosuppressive effects in order to get the most out of ICD mediated treatment.
